# Maladie dermatophytique de revelation tardive

**DOI:** 10.11604/pamj.2016.24.194.6509

**Published:** 2016-07-07

**Authors:** Christelle Natacha Ebongo Aboutou, Fouzia Hali, Soumya Chihab

**Affiliations:** 1 Service de Dermatologie CHU Ibn Rochd de Casablanca, Maroc

**Keywords:** Maladie dermatophytique, revelation tardive, peau, viscères, Dermatophytic disease, late onset, skin, viscera

## Abstract

La maladie dermatophytique, décrite pour la première fois en 1959 par Hadida et Schousboe, est une infection dermatophytique chronique de la peau et des viscères. C'est une maladie rare principalement décrite au Maghreb. Les études immunologiques ont permis de mettre en évidence un déficit de l'immunité cellulaire à transmission autosomale récessive responsable d'un état de tolérance vis-à-vis du dermatophyte. Les premiers signes de cette pathologie surviennent généralement pendant l'enfance. Notre patient présente depuis l'âge de 50 ans une pachyonychie de tous les ongles et des lésions érythémato-squameuses circinées et prurigineuses généralisées à tout le tégument auxquelles se sont progressivement ajoutées une alopécie et une dépilation de toutes les aires pilaires, une kératodermie palmo-plantaire ainsi que des adénopathies axillaires et inguinales bilatérales. Tricophyton violaceum a été isolé sur les ongles. Aucun déficit immunitaire n'a été retrouvé ni atteinte viscérale dans la limite des bilans réalisés. L'évolution est marquée par des améliorations transitoires, la résistance des adénopathies et des atteintes phanériennes ainsi que les multiples rechutes malgré la mise sous griséofulvine. La maladie dermatophytique est une maladie grave mettant en jeu le pronostic vital du fait de l'évolution inexorable vers les atteintes viscérales. L'amélioration du statut immunitaire associée au traitement antifongique pourrait être la meilleure thérapeutique.

## Abstract (en)

Dermatophytic disease, described for the first time in 1959 by Hadida and Schousboe, is a chronic dermatophyte infection of the skin and viscera. It is a rare disease occurring mainly in Maghreb. Immunological studies have highlighted a deficit of cellular immunity with autosomal recessive transmission responsible for tolerance to dermatophyte. The first signs of this disease usually occur during childhood. Our patient suffered from pachyonychia affecting all his nails and erythematous, circinate, pruritic, scaly lesions occurring in all his seed coat from the age of 50 years. These disorders were gradually followed by alopecia and hair removal of all body hairy areas, palmoplantar keratoderma as well as bilateral axillary and inguinal adenopathies. Tricophyton violaceum was isolated from patient’s nails. No immune deficiency was found or visceral involvement within the limits of the assessments made. The evolution was marked by transient improvements, resistance of adenopathies and skin appendage involvement as well as by multiple relapses despite griseofulvin therapy. Dermatophytic disease is a serious life-threatening disease due to its inexorable evolution toward visceral involvement. The improvement of patient’s immune system associated with antifungal therapy may be the best treatment.

## Introduction

La maladie dermatophytique, décrite par Hadida et Schousboe en 1959, est une dermatophytie généralisée et chronique de la peau et des phanères avec des localisations secondaires dermohypodermiques, ganglionnaires et viscérales survenant sur un terrain particulier [1]. C'est une pathologie rare avec une cinquantaine de cas seulement rapportés dans la littérature [2]. Elle est essentiellement décrite en Afrique du Nord [2]. D'une part les cas familiaux et la consanguinité font évoquer une transmission autosomique récessive et d'autre part la chronicité et la résistance partielle aux antifongiques font évoquer un déficit immunitaire cellulaire [2, 3]. Elle atteint surtout le sujet de sexe masculin et les premiers signes apparaissent généralement pendant l'enfance [2]. Après l'Algérie, le Maroc est le deuxième pays où le plus grand nombre de cas ont été rapportés [4]. Nous rapportons un cas à début tardif.

## Patient et observation

Il s'agit de Mr. LBA, 65 ans, marié et père de 4 enfants, sans antécédent pathologique particulier, pas de notion de consanguinité des parents ni de cas similaire dans la famille et pas de notion de teignes ou d'infections dermatophytiques récidivantes dans l'enfance. Il présente depuis 14 ans des lésions érythémato-squameuses, circinées, prurigineuses et d'extension centrifuge s'étant progressivement généralisées à tout le tégument ainsi qu'une pachyonychie de tous les ongles. Ceci évolue dans un contexte de conservation de l'état général. L'examen mycologique a isolé Trichophytum violaceum et les deux premières biopsies cutanées réalisées étaient non spécifiques. Il est alors mis sous griséofulvine avec bonne évolution. Devant les rechutes systématiques à l'arrêt du traitement de nouveaux prélèvements de squames cutanées et des ongles pour étude mycologique ainsi qu'une nouvelle biopsie cutanée sont réalisées huit ans plus tard mettant en évidence des filaments mycéliens à l'examen direct mais à culture négative. Les tests de dépistage des hépatites B et C, du VIH ainsi que de la syphilis se sont tous révélés négatifs. Le diagnostic de maladie dermatophytique est alors retenu et le patient mis sous griséofulvine à la dose d'1g/j à maintenir toute la vie. Mais à cause de difficultés financières le patient ne peut suivre le traitement et est perdu de vue.

**Figure 1:**
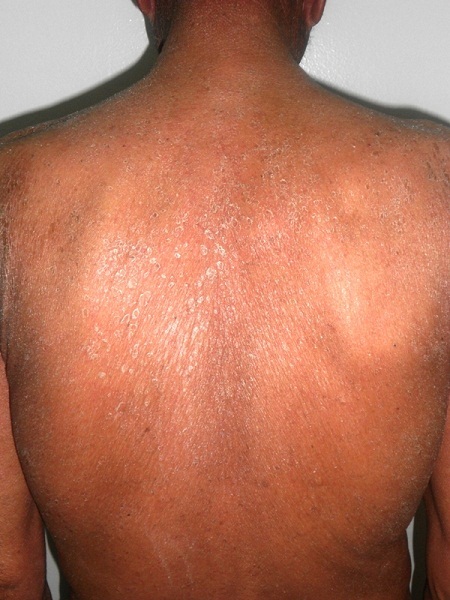
Lésions érythémato-squameuses

**Figure 2:**
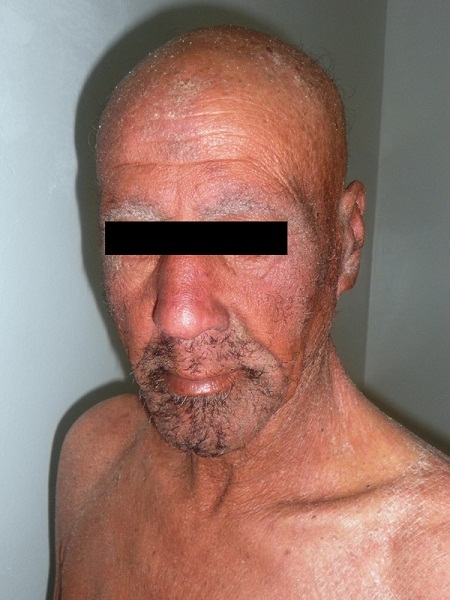
Alopécie et dépilation sourcils et barbe

**Figure 3:**
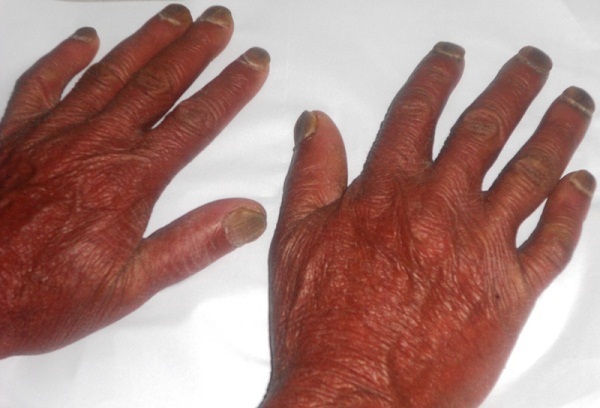
Pachyonychie des ongles des mains

**Figure 4:**
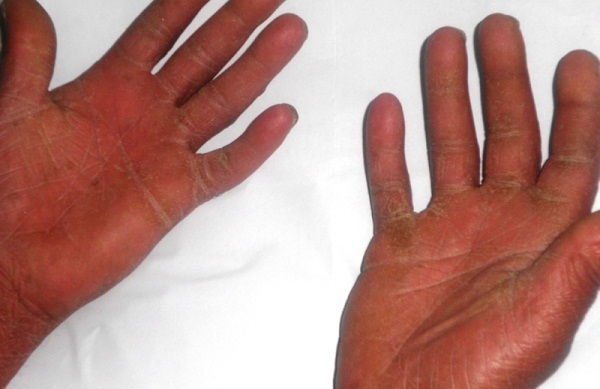
Kératodermie palmaire

Neuf ans plus tard l'évolution est marquée par l'installation d'une érythrodermie sèche (Figure 1) avec kératodermie palmo-plantaire et pachyonychie des vingt ongles (Figure 2, Figure 3) associées à de volumineuses adénopathies axillaires et inguinales bilatérales douloureuses ainsi qu'à une alopécie érythémato-squameuse et une dépilation totale (Figure 4). L'examen direct des squames cutanées et des ongles ainsi que la biopsie cutanée ont à nouveau mis en évidence des filaments mycéliens, mais la culture a été négative. L'hémogramme était sans anomalie et la sérologie VIH négative. Un bilan d'extension à la recherche d'atteintes viscérales est réalisé: une radiographie standard du thorax et une échographie abdomino-pelvienne qui ont été toutes deux normales. Le patient a été maintenu sous griséofulvine à 1g/j et traitement local notamment antiseptiques et antifongiques locaux avec éducation sur l'importance du maintien du traitement. Une lettre a également été adressée à l'hôpital périphérique afin que celui-ci lui procure son traitement. L'évolution est marquée par la régression des lésions cutanées mais persistance de la pachyonychie et des adénopathies.

## Discussion

La maladie dermatophytique est une pathologie rare, classée parmi les maladies orphelines. Il s'agit d'une dermatophytose cutanéoviscérale chronique. Depuis son individualisation en 1959 par Hadida et Shousboe, une cinquantaine d'observations ont été rapportées provenant presque exclusivement du Maghreb [2, 4, 5]. L'Algérie est le pays où le maximum de cas a été rapporté suivi du Maroc. On note une nette prédominance masculine. Elle atteint principalement l'adulte jeune mais débute généralement à l'enfance [2]. Chez notre patient les premiers signes sont apparus à l'âge de 50 ans ce qui est rare (6,7% après 50 ans) [2]. La physiopathologie reste mal élucidée mais un déficit immunitaire à transmission récessive affectant l'immunité cellulaire serait à l'origine d'un état de tolérance vis-à-vis des dermatophytes. Si les notions de consanguinité et des cas familiaux sont fréquemment retrouvés ils ne sont pas systématiques comme le démontre notre cas.

Sur le plan clinique la maladie dermatophytique débute le plus souvent par une teigne du cuir chevelu récidivante ou par une atteinte de la peau glabre. Les formes évoluées peuvent revêtir plusieurs aspects: des plaques alopéciques pouvant intéresser toutes les aires pilaires, des lésions érythémato-squameuses pouvant former une érythrodermie, un prurit intense, des papulonodules voire des abcès sous-cutanés, une kératodermie palmo-plantaire, une pachyonychie intéressant tous les vingt ongles [2, 4, 6]. Les muqueuses sont épargnées. Des adénopathies sont retrouvées chez plus de la moitié des patients, toutes les aires ganglionnaires pouvant être touchées mais l'atteinte des aires profondes est rare et tardive [2]. À un stade plus tardif, les lésions peuvent se propager et gagner les organes profonds (os, cerveau …) [1, 7]. Ces différentes atteintes sont caractérisées par des résistances ou des rechutes fréquentes surtout à l'arrêt du traitement comme chez notre patient. A l'histologie, l'élément le plus évocateur est le granulome retrouvé dans pratiquement toutes les lésions profondes (tubercules, nodules, nodosités hypodermiques, ganglions, etc.) Une organisation folliculaire très tuberculoïde peut être retrouvée au sein de ce granulome, centrée alors par des foyers de nécroses riches en filaments mycéliens. A ce stade la maladie dermatophytique peut être prise à tord pour une tuberculose.

L'examen direct est souvent positif aussi bien au niveau des prélèvements superficiels que profonds et l'espèce la plus incriminée est T. violaceum qui est le dermatophyte le plus fréquemment isolé dans les teignes au Maghreb [8]. Chez notre patient il avait été retrouvé lors du tout premier prélèvement mais par la suite à cause du traitement fongique initié les cultures s'étaient toutes révélées négatives.

L'évolution de la maladie dermatophytique est en moyenne de 25 ans [5]. Actuellement il n'existe pas de schéma thérapeutique codifié et les différents traitements utilisés ne permettent qu'un contrôle partiel de la maladie. L'amélioration du statut immunitaire associée au traitement antifongique pourrait être la meilleure thérapeutique. La griséofulvine est l'antifongique le plus administré souvent associée aux traitements locaux. Elle est prescrite à la dose de 1 g/j. Cependant des molécules plus récentes comme la terbinafine, le fluconazole et l'itraconazole se sont révélées plus efficaces mais bien plus onéreuses. Mais comme dans notre cas, les antifongiques, même les plus récents, ne sont pas suffisants à eux seuls pour obtenir une guérison complète. En effet une intervention sur l'immunité est indispensable et pour cette raison l'association entre l'interféron gamma et les antifongiques reste la meilleure option thérapeutique bien que assez onéreuse. Le traitement antifongique devant être maintenu pendant plusieurs années, une surveillance hépatique est indispensable à cause de l'hépatotoxicité de ces molécules. La maladie dermatophytique met non seulement en jeu la vie sociale des patients par le caractère affichant des lésions mais engage aussi le pronostic vital par l'évolution inexorable vers les atteintes profondes et viscérales.

## Conclusion

La maladie dermatophytique est une maladie rare. Si la physiopathologie exacte reste inconnue, un déficit de l'immunité cellulaire à transmission récessive en est responsable. Si les formes à manifestation précoce sont les plus fréquentes, des formes à début tardif restent possibles. Les manifestations cliniques sont multiples et variables et l'histologie et la mycologie permettent de confirmer le diagnostic. La résistance au traitement antifongique et l'évolution inexorable vers les atteintes viscérales en font une maladie grave au pronostic sombre.
